# IgG4-related focal retroperitoneal fibrosis in ureter suggestive of colon cancer recurrence and resected laparoscopically: a case report

**DOI:** 10.1186/s40792-020-00964-0

**Published:** 2020-08-03

**Authors:** Tomoyuki Ueki, Toru Miyake, Mitsuhiro Narita, Masatsugu Kojima, Sachiko Kaida, Hiroya Iida, Tomoharu Shimizu, Masaji Tani

**Affiliations:** 1 Department of Surgery, Shiga University of Medical Science, Tsukinowa-cho, Seta, Otsu-shi, Shiga, 520-2192 Japan; 2 Department of Urology, Shiga University of Medical Science, Tsukinowa-cho, Seta, Otsu-shi, Shiga, 520-2192 Japan

**Keywords:** IgG4-related disease, IgG4-related focal retroperitoneal fibrosis, Laparoscopic surgery, Ureter

## Abstract

**Background:**

Immunoglobulin G4-related disease (IgG4-RD) is a novel disease concept of unknown cause that is characterized by abundant infiltration of IgG4-positive cells, mass-forming lesions, and elevated serum IgG4 levels. The infiltration of IgG4-positive plasma cells and lymphocytes causes swelling, inflammation, fibrosis, and obliterative phlebitis in multiple organs. On the other hand, IgG4-RD occurring in the ureters has rarely been reported. To our knowledge, this is the first report of laparoscopic partial ureteral resection for IgG4-related focal retroperitoneal fibrosis in a ureter with suspected colon cancer recurrence.

**Case presentation:**

A 72-year-old man with a history of sigmoid colon cancer visited Shiga University of Medical Science Hospital for regular follow-up in December 2019. Enhanced abdominal computed tomography revealed a mass involving the left ureter. Furthermore, fluorine-18 fluorodeoxyglucose positron emission tomography showed significant accumulation of fluorodeoxyglucose uptake in the same region. Due to the possibility of colon cancer recurrence, a laparoscopic excisional biopsy with partial ureteral resection was performed. Histologically, IgG4-positive plasma cell infiltration exceeding 10 cells per high-power field and a high ratio of IgG4-positive/IgG-positive cells exceeding 40% were observed. The postoperative serum IgG4 level was 384 mg/dL. With the application of these findings to the diagnostic algorithm in the comprehensive diagnostic criteria for IgG4-RD, the mass-forming lesion was diagnosed as definitive IgG4-related focal retroperitoneal fibrosis.

**Conclusions:**

IgG4-RD should be considered in the differential diagnosis of retroperitoneal lesions. Moreover, laparoscopic surgery may be useful for making the diagnosis in difficult-to-biopsy cases.

## Background

Immunoglobulin G4-related disease (IgG4-RD) is a novel systemic disease of unknown cause that is characterized by IgG4-positive plasma cell and lymphocyte infiltration histologically and elevated serum IgG4 levels. This disease, first described by Hamano et al. [1], affects various organs including the salivary glands, pancreas, liver, bile duct, lungs, thyroid, kidneys, prostate, retroperitoneum, lymph nodes, and breasts. Focal retroperitoneal fibrosis, a type of IgG4-RD, is characterized by thickening of the abdominal aortic adventitia and soft tissue around the ureter [2]. On the other hand, the differential diagnosis from secondary retroperitoneal fibrosis caused by various diseases such as malignancy and infection often becomes clinically problematic because biopsy is difficult in many cases. Here, we report a case of IgG4-related focal retroperitoneal fibrosis in a ureter suggestive of colon cancer recurrence that was diagnosed by laparoscopic resection.

## Case presentation

A 72-year-old man visited Shiga University of Medical Science Hospital for regular follow-up of sigmoid colon cancer in December 2019. He had a medical history of undergoing an appendectomy at age 10 and laparoscopic sigmoidectomy for sigmoid colon cancer (pStage I) at age 70. The histopathological diagnosis of the colon cancer was moderately differentiated tubular adenocarcinoma and tumor invasion to the submucosa with venous invasion. His father died of gastric cancer. Enhanced abdominal computed tomography (CT) revealed a mass involving the left ureter that had grown slowly since the previous CT. He underwent further examinations for the diagnosis. A physical examination revealed no abdominal pain or palpable mass. Additionally, no enlarged lymph nodes were palpable in the cervical, axillary, or inguinal regions. Laboratory data included elevated lactate dehydrogenase (239 U/L), creatinine (1.14 mg/dL), and carbohydrate antigen 19-9 (41 U/mL) levels. Other serum biochemical parameters and tumor markers were within the normal ranges. Urine cytology showed no atypical cells (class II).

**Fig. 1:**
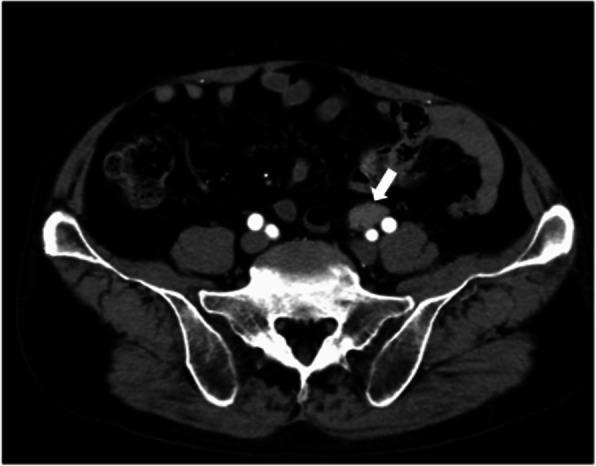
Enhanced abdominal computed tomography (CT) findings. Enhanced abdominal CT showed a 30-mm soft-tissue mass involving the left ureter in front of the bifurcation of the left common iliac artery (arrow)

**Fig. 2:**
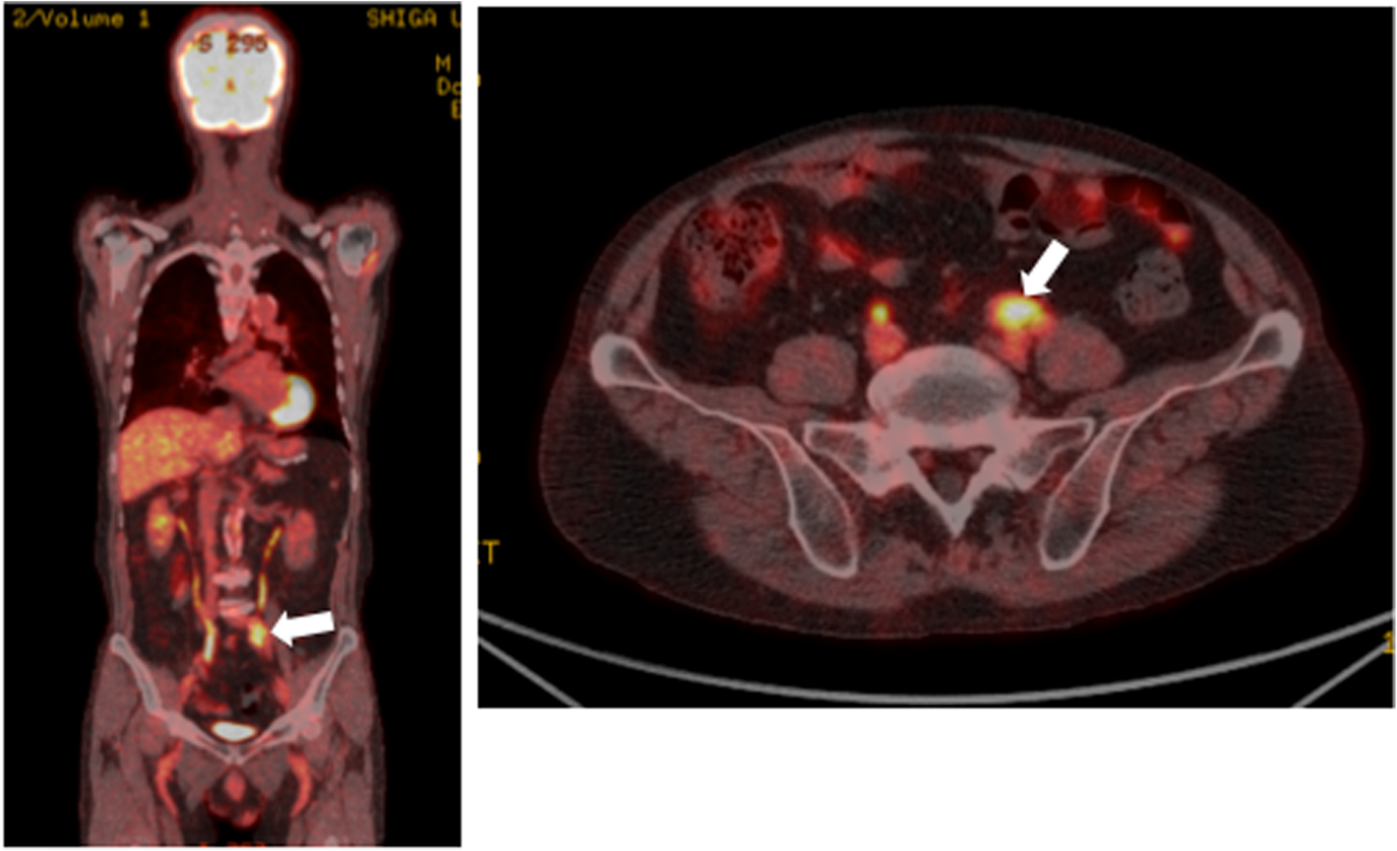
Fluorine-18 fluorodeoxyglucose (FDG) positron emission tomography CT (PET-CT) findings. FDG-PET-CT showed significant accumulation of FDG uptake in the same region (maximum standardized uptake value, 8.93; arrow)

Enhanced abdominal CT showed a 30-mm soft-tissue mass involving the left ureter in front of the bifurcation of the left common iliac artery but no enlarged lymph nodes, hydronephrosis, or other abnormal findings (Fig. 1). The mass had grown from 16.7 × 10.3 mm to 30 × 13.7 mm in 17 months. Fluorine-18 fluorodeoxyglucose (FDG) positron emission tomography CT (PET-CT) showed significant accumulation of FDG uptake in the same region (maximum standardized uptake value, 8.93; Fig. 2). Significant FDG uptake was not observed at any of the other sites. Ureterography and colonoscopy showed no abnormal findings.

**Fig. 3:**
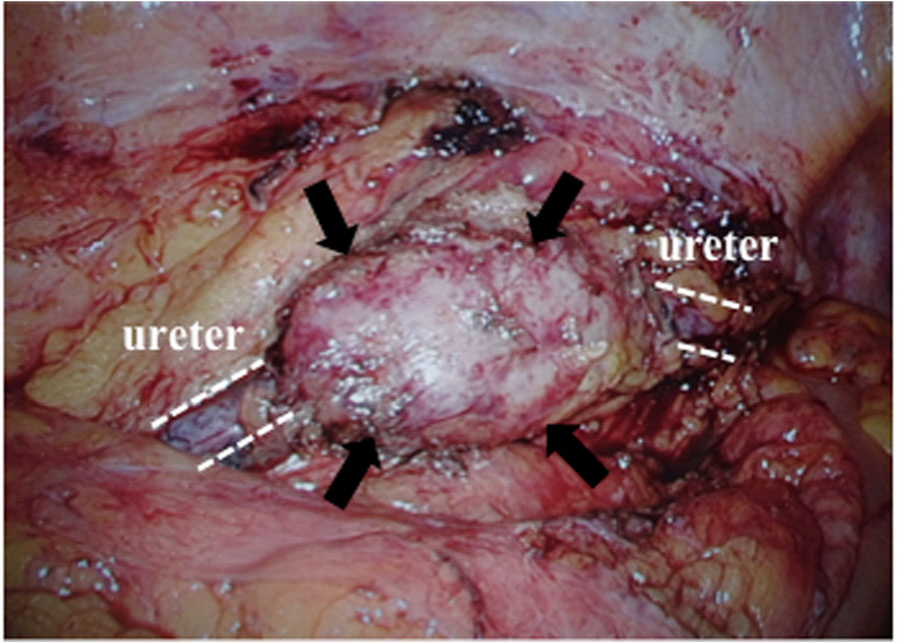
Intraoperative findings. The mass around the left ureter was observed (mass; black arrow, ureter; white dotted line)

Due to the possible lymph node recurrence or peritoneal seeding of colon cancer indicated by the slow growth and significant FDG uptake, we performed a laparoscopic excisional biopsy. Under general anesthesia, the patient was placed in the lithotomy–Trendelenburg position. First, a ureteral stent was inserted in the left ureter radiologically using a guide wire. The endoscopist stood to the upper right of the patient, the surgeon stood near the surgeon’s right hand, and the assistant stood opposite the surgeon. A transumbilical skin and fascia incision was performed. The peritoneum was opened, and a 12-mm trocar was introduced. Under pneumoperitoneum, a 12-mm trocar was placed in the lower right abdomen, and 5-mm trocars were placed in the bilateral lower and upper abdomen. We dissected and mobilized the left-sided colon using the medial-to-lateral approach in Toldt’s space. After recognizing that the left ureter was involved in the mass-forming lesion in front of the bifurcation of the left common iliac artery, we mobilized the left ureter throughout the whole circumference of the mass-forming lesion (Fig. 3). After removing the stent, we dissected the proximal and distal ureter of the mass-forming lesion and removed the specimen from the umbilical port site. The intraoperative frozen section examination diagnosed the mass as a benign lymphoproliferative lesion. Two 4-0 Vicryl® sutures were used to form a running anastomosis. After finishing the anastomosis of the posterior ureteral wall, a 6Fr 26-cm double-J catheter was inserted laparoscopically. After ensuring the double-J catheter into the renal pelvis, running suture anastomosis of the anterior ureteral wall was completed. The total operative blood loss was 8 mL, and the operative time was 321 min. The postoperative course was uneventful, and he was discharged on postoperative day 6. After 3 months of follow-up, he had not developed any recurrent lesions or any other IgG4-RD, including autoimmune pancreatitis.

**Fig. 4:**
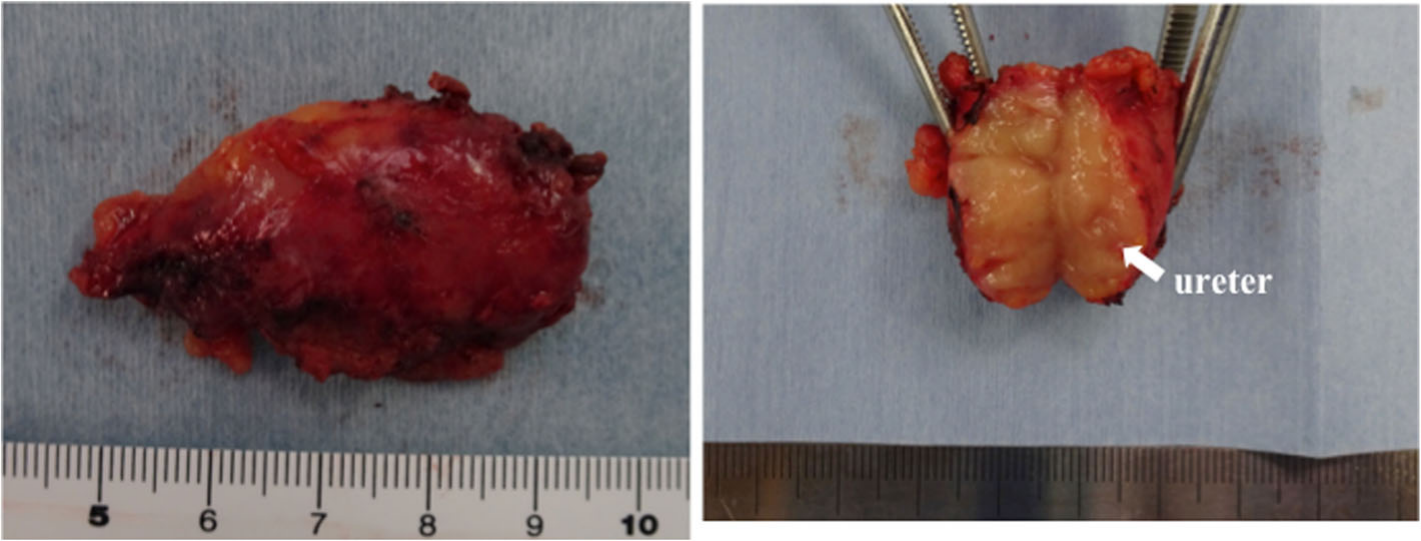
Macroscopic findings of the resected specimen. An elastic and soft mass similar to a swollen lymph node with a lumen structure (ureter) was detected (arrow)

**Fig. 5:**
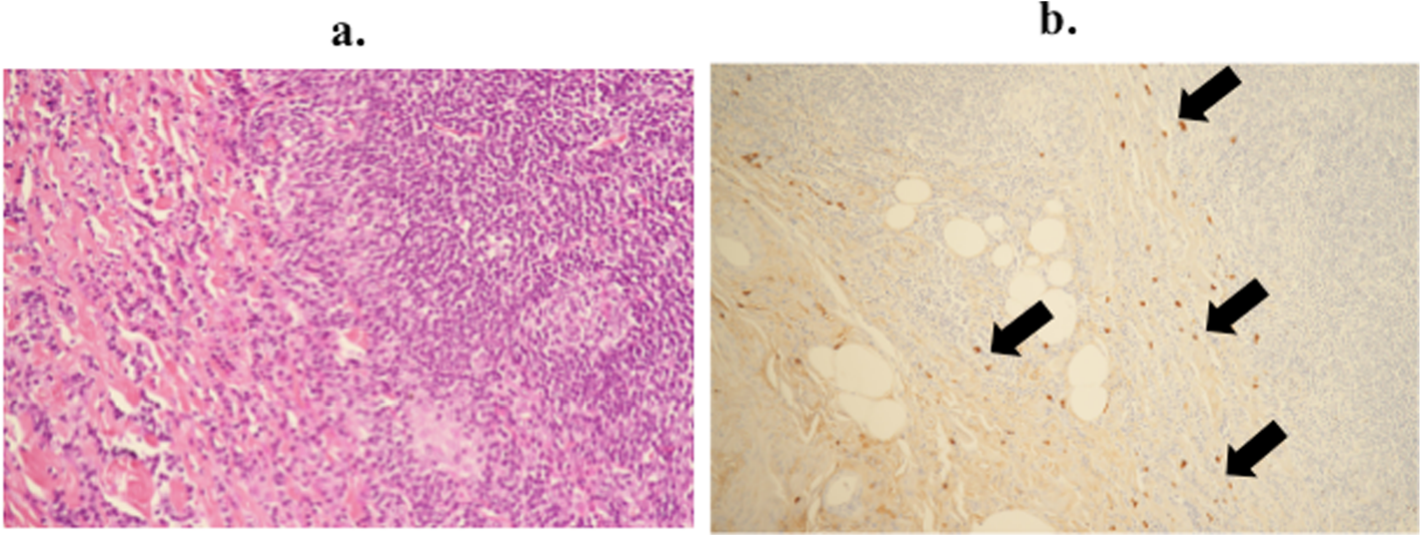
Histopathological findings. **a** Hematoxylin and eosin staining showed abundant infiltration of lymphocytes with lymphoid follicles around the left ureter and peripheral proliferation of collagenous fibers (× 200). **b** Immunohistochemical staining showed that the mean IgG4-positive plasma cell count was 50 per high-power field, and the ratio of IgG4-positive/IgG-positive cells was 54% (IgG4, immunoperoxidase, × 100; arrow)

Macroscopically, an elastic and soft mass similar to a swollen lymph node with a lumen structure was detected (Fig. 4). A histological examination showed abundant infiltration of lymphocytes with lymphoid follicles around the left ureter and peripheral proliferation of collagenous fibers. Immunohistochemically, the mean IgG4-positive plasma cell count was 50 per high-power field (HPF), and the ratio of IgG4-positive/IgG-positive cells was 54% (Fig. 5). The postoperative serum IgG4 level was 384 mg/dL. Applying these findings to the diagnostic algorithm in the comprehensive diagnostic criteria for IgG4-RD [3], the mass-forming lesion was definitively diagnosed as IgG4-related focal retroperitoneal fibrosis.

## Discussion

IgG4-RD is a novel disease characterized by the abundant infiltration of IgG4-positive cells, mass-forming lesions, and elevated serum IgG4 levels. This disease was first described by Hamano et al., who reported elevated IgG4 levels in patients with autoimmune pancreatitis [1]. Clinically, IgG4-RD shows diffuse/localized swelling or mass formation in single or multiple organs, including the salivary glands, pancreas, liver, kidneys, retroperitoneum, and lymph nodes. The infiltration of IgG4-positive plasma cells and lymphocytes causes swelling, inflammation, fibrosis, and obliterative phlebitis in multiple organs. IgG4-RD was predominant in elderly men, affecting about 90%, particularly in the kidneys and retroperitoneum [2, 4–6].

The diagnosis of IgG4-RD is based on clinical and histological features. In Japan, the Ministry of Health, Labour and Welfare released the Comprehensive Diagnostic Criteria for IgG4-RD in 2011 [3]. In our case, the enhanced abdominal CT showed a localized swollen mass around the left ureter and the serum IgG4 level was over 135 mg/dL. Furthermore, the histological examination showed abundant lymphocytic and plasmacytic infiltration with an increased number of IgG4-positive plasma cells (> 10/HPF and a > 40% ratio of IgG4-positive/IgG-positive cells). Based on these features, our case was diagnosed as IgG4-related focal retroperitoneal fibrosis.

On the other hand, secondary retroperitoneal fibrosis caused by drugs, radiation, bleeding, malignancy, and infection had to be considered as a differential disease in the preoperative diagnosis. In our case, these factors were excluded based on symptoms, medical history, and examination findings. The ureteral tumor was also excluded because there were no abnormal findings on the preoperative ureterography or malignant cells in the urine cytology. However, in our case, it was difficult to completely distinguish malignant tumors from benign lesions. No specific symptoms associated with the mass-forming lesion were observed, but colon cancer recurrence was possible considering the slow growth and significant FDG uptake. Therefore, we performed laparoscopic resection to enable the diagnosis and treatment. Retrospectively, serum IgG4 level should be measured before the preoperative diagnosis since IgG4-RD is a differential disease. Although performing a biopsy for the definitive diagnosis is often difficult when retroperitoneal fibrosis is close to various organs, laparoscopic surgery enabled minimally invasive excisional biopsy in this case.

IgG4-RD treatment remains controversial. Yoshino et al. reported that corticosteroid therapy was effective for retroperitoneal fibrosis with elevated serum IgG4 level, and clinical symptoms improved as serum IgG4 level decreased [7]. However, there are no definitive criteria for corticosteroid dose or duration, and follow-up without treatment is often provided in asymptomatic cases [8]. In our case, the patient received no corticosteroid therapy after surgery because he was asymptomatic and concerned about the side effects of long-term corticosteroid use.

On the other hand, Wallace et al. reported that a history of malignancy might be associated with subsequent IgG4-RD development [9]. One possibility is that treatments for malignancy, including chemotherapy, cause immunodeficiency and lead to IgG4-RD. In our case, due to no postoperative adjuvant chemotherapy and a long period after sigmoidectomy, we thought that the sigmoid colon cancer was not significantly associated with the mass-forming lesion. The histopathological examination of the sigmoid colon cancer performed retrospectively revealed IgG4-positive plasma cells (> 10/HPF). However, the significance is controversial, because IgG4-positive cells were similarly detected in the normal mucosa.

Moreover, Hamano et al. reported the probability of developing IgG4-RD including autoimmune pancreatitis during the clinical course of IgG4-related retroperitoneal fibrosis [1]. Our patient had no specific symptoms associated with autoimmune pancreatitis, such as back pain, general malaise, body weight loss, or jaundice. Furthermore, on enhanced abdominal CT, no pancreatic enlargement or main pancreatic duct narrowing was observed. However, for the above-mentioned reason, he required continual monitoring for the occurrence of other organ lesions.

To our knowledge, the published literature concerning IgG4-RD occurring in ureters is rare, with only 13 cases found globally in our survey. All cases including ours are summarized in Table 1 [10–18], which shows that 11 (78.6%) were men and 3 (21.4%) were women. The median patient age was 74 years (range, 39–84 years). The median serum IgG4 level was 384 mg/dL (range, 206–965) in 9 cases. All cases were over the standard value. A nephroureterectomy was performed in 7 cases versus a partial ureteral resection in 7 cases. Three cases were treated laparoscopically. Two patients underwent laparoscopic nephroureterectomy, while our patient underwent laparoscopic partial ureteral resection. This is the first report of laparoscopic partial ureteral resection for IgG4-related focal retroperitoneal fibrosis in a ureter with suspected colon cancer recurrence.

**Table 1:** Summary of IgG4-related disease in the ureter

Case	Author	Age	Sex	Clinical diagnosis	IgG4 (mg/dL)	Hydronephrosis	AIP	Operation	Laparoscopy	Steroid
1	Hamano et al. [10]	60	M	Cancer	265	+	+	Nephroureterectomy	−	40 mg
2	Hamano et al. [10]	74	M	Cancer	965	+	+	Partial ureteral resection	−	40 mg
3	Kamisawa et al. [11]	75	M	Cancer	240	+	+	Partial ureteral resection	−	Not described
4	Bessho et al. [12]	74	M	Cancer	412	+	−	Nephroureterectomy	−	Not described
5	Kim et al. [13]	45	M	Cancer	–	−	−	Nephroureterectomy	−	−
6	Kim et al. [13]	47	M	Cancer	855	+	−	Partial ureteral resection	−	−
7	Kim et al. [13]	84	F	Cancer	–	+	−	Nephroureterectomy	−	−
8	Abe et al. [14]	39	M	Cancer	233	+	−	Partial ureteral resection	−	Not described
9	Nomura et al. [15]	79	F	Cancer	206	+	−	Nephroureterectomy	+	−
10	Marand et al. [16]	77	M	Cancer	–	−	−	Partial ureteral resection	−	Not described
11	Marand et al. [16]	82	F	Cancer	–	+	−	Partial ureteral resection	−	Not described
12	Lei WH et al. [17]	66	M	Cancer	913	+	−	Nephroureterectomy	+	60 mg
13	Zhong W et al. [18]	64	M	Cancer	–	+	−	Nephroureterectomy	−	Not described
14	Our case	72	M	Cancer	384	−	−	Partial ureteral resection	+	−

## Conclusion

Here, we reported a case of IgG4-related focal retroperitoneal fibrosis in a ureter with suspected colon cancer recurrence that was resected by laparoscopic surgery. IgG4-RD should be considered in the differential diagnosis of retroperitoneal lesions. Moreover, laparoscopic resection could be useful for enabling the diagnosis of difficult-to-biopsy cases.

## Data Availability

Not applicable

## Abbreviations

AIP: Autoimmune pancreatitis
CT: Computed tomography
FDG: Fluorine-18 fluorodeoxyglucose
HPF: High-power field
IgG4-RD: Immunoglobulin G4-related disease
PET-CT: Positron emission tomography CT
